# Epigenetic changes in localized gastric cancer: the role of RUNX3 in tumor progression and the immune microenvironment

**DOI:** 10.18632/oncotarget.11520

**Published:** 2016-08-23

**Authors:** Marta Jessica Llorca-Cardeñosa, Tania Fleitas, Maider Ibarrola-Villava, María Peña-Chilet, Cristina Mongort, Carolina Martinez-Ciarpaglini, Lara Navarro, Valentina Gambardella, Josefa Castillo, Susana Roselló, Samuel Navarro, Gloria Ribas, Andrés Cervantes

**Affiliations:** ^1^ Medical Oncology, Biomedical Research Institute INCLIVA, University of Valencia, Valencia, Spain; ^2^ Department of Pathology, Biomedical Research Institute INCLIVA, University of Valencia, Valencia, Spain

**Keywords:** RUNX3, ARID1A, gastric cancer, gene methylation, immune microenvironment

## Abstract

Gastric cancer (GC) pathogenesis involves genetic, epigenetic and environmental factors. Epigenetic alterations, such as DNA methylation are considered pivotal in the inactivation of tumor-related genes. We assessed a methylation panel of 5 genes to study their association to GC progression and microsatellite instability (MSI), and studied the role of *RUNX3* in GC pathogenesis and the tumor immune microenvironment.

The methylation status of 47 promoter-CpG islands was studied through MALDI-TOF mass spectrometry analysis in 35 Microsatellite stable (MSS) GC, 26 MSI, and 18 cancer-free samples (CFS), and 6 MSS GC and 4 MSI GC cell lines. We also studied RUNX3 expression by immunohistochemistry (IHC) in 40 samples, and validated differences in methylation levels between tumor, normal, and immune tissue in 14 additional samples.

Unsupervised hierarchical clustering of methylation levels revealed no distinct subgroups between MSI and MSS samples or cell lines. CFSs clustered together showing higher levels of *RUNX3* methylation compared to GC samples. *RUNX3* showed protein silencing in cancer and normal mucosa, compared to inflammatory peritumoural infiltrate in almost all cases, showing a non-lymphocytic predominant pattern and being correlated with epigenetic silencing.

Our results show aberrant promoter's methylation in *APC, CDH1, CDKN2A,* MLH1 and *RUNX3* associated with GC, as well as a non-lymphocytic predominant infiltrate with high expression of *RUNX3*. Deep study of *RUNX3* inflammation signaling could help in understanding inflammation and immune activation in the tumor microenvironment.

## INTRODUCTION

Gastric cancer (GC) has high incidence and mortality and is among the most common malignancies: in 2012 it was the second-leading cause of cancer-related deaths worldwide [1]. The roles played by genetic and epigenetic alterations in causing GC are being increasingly recognized, but currently only *HER2* overexpression is used as a marker for target-based therapy [2]. Thus, comprehensive molecular characterization of GC is urgently needed in order to better stratify patients and personalize their treatments [3–5].

Epigenetic alterations, such as CpG island DNA methylation, are involved in gastric carcinogenesis [6], and promoter methylation is considered to be one of the key processes involved in inactivating tumor suppressor-related genes. Epigenetic inactivation of several genes has recently been related with GC progression [6–8], and includes genes involved in cell cycle regulation (*CDKN2A*), DNA repair (*MLH1*), cell adhesion/invasion/migration (*CDH1*), STAT and Wnt pathways (*APC*), transcriptional regulation (*RUNX3*), and many others. Furthermore, aberrant methylation of these genes has been previously related to the CpG island methylator phenotype (CIMP) [9–19] which was first described in colorectal cancer (CRC) and refers to the concurrence of hypermethylation in multiple genes [14, 17]. Despite the presence of the CIMP phenotype in GC having been reported by many scientists, data regarding its prognostic value for this cancer remains controversial [9–12, 20]. Moreover, according to the Cancer Genome Atlas Research Network, the CIMP phenotype is related to the microsatellite instability (MSI) GC subgroup and is also associated with female gender, antral tumor location, better survival rates, mutations in *ARID1A*, *KRAS*, *HER2,* and PIK3/PTEN/mTOR pathway involvement [5].

Additionally, the *RUNX3* transcription factor, poorly qualified as a tumor suppressor gene (TSG) [21–23], has been associated with early inflammatory, pre-neoplastic, and tumor stages [24] as well as with chronic *H. pylori* infection [15, 25], which is known to lead to inflammation in gastric tissue and may induce atrophy, dysplasia, and metaplasia [26]. During chronic inflammation genetic and epigenetic changes work in concert to alter important pathways involved in normal cellular function, and hence accelerate inflammation-associated cancer development [27].

Thus, we assessed the association of a panel of five marker genes to study their association to MSI subgroup, CIMP-phenotype, and GC-progression, as well as the role of *RUNX3* as a conflicting TSG [21–23] compared to a known TSG, *ARID1A,* in GC pathogenesis, *H. pylori* infection, MSI, and the tumor immune microenvironment.

## RESULTS

### Gene methylation panel analysis

Clinicopathological characteristics such as age, sex, tumor location, histology, tumor grade (based on the TNM classification system for malignant tumors, 7th edition), *HER2*, *HER3*, *cMET*, *ARID1A* expression, microsatellite status and treatments administered to patients with GC included in the initial methylation panel (*n* = 61) are shown in Table 1.

**Table 1:** Clinicopathological characteristics of samples included in the initial methylation panel (*N* = 61)

	*n* (*N* = 61)
**Mean age in years (*SD*)**	71.03 (14.12)
**Gender**	
Male	30
Female	31
**Localization**	
Antrum	25
Body	15
GEJ	9
Cardia	6
Fundus	5
Gastric stump	1
**Lauren Classification**	
Intestinal	40
Diffuse	15
Mixed	6
**Stage**	
I	11
II	33
III	8
IV	4
Unknown	5
**Treatment**	
No	38
XELOX	19
FOLFOX	1
Other	3
**Microsatellite Instability**	
MSI	26
MSS	34
Unknown	1
**HER2 amplification**	
Her2+	13
Her2–	48
**HER3 amplification**	
Her3+	13
Her3–	47
unknown	1
**cMET amplification**	
cMet+	13
cMet–	48
**ARID1A Loss**	
Arid1a wt	37
Arid1a –	24

GEJ, gastro-esophageal junction; XELOX, capecitabine plus oxaliplatin; FOLFOX, folinic acid, fluorouracil and oxaliplatin; MSI, microsatellite instability; MSS, microsatellite stability; wt, wild-type.

**Figure 1:**
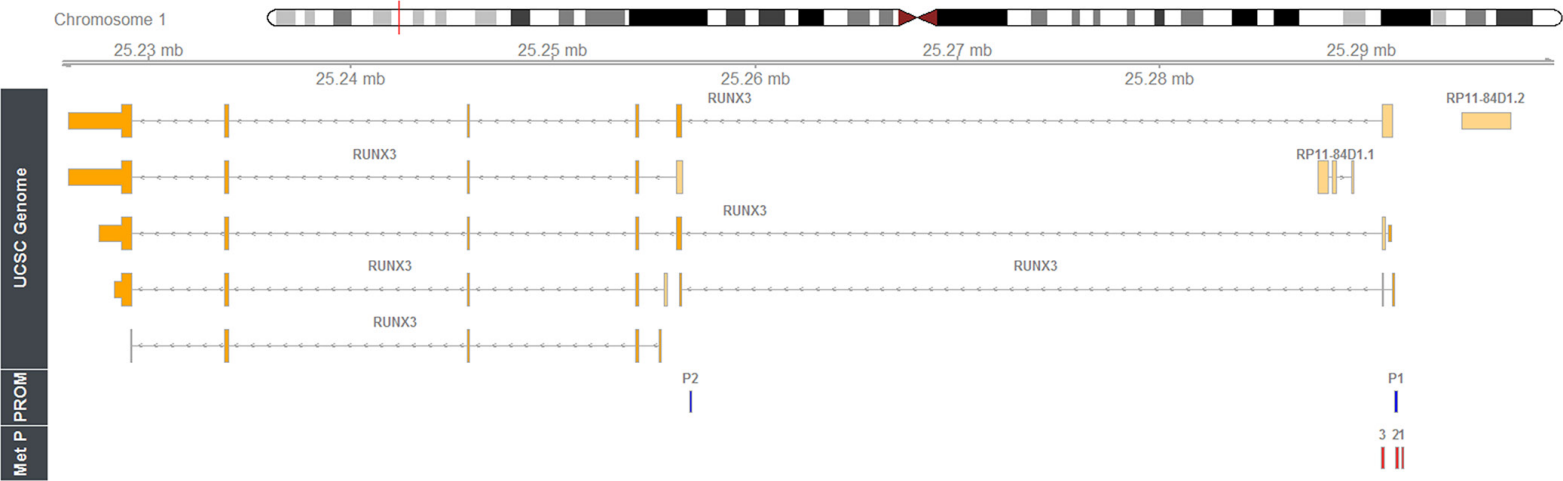
Mapping of the methylation amplicons studied within human RUNX3 gene Rbioconductor/Rstudio and package Gviz (REF) has been used to show genomic data for human RUNX3 on chromosome 1p36 (using as genomic source GRCh37 Hg19; http://genome.ucsc.edu/). Relative locations of P1 and P2 promoter regions and intron/exon structures of the derived transcripts are shown. Browser tracks show locations of RUNX3 methylation amplicons; 1 = RUNX3.4, 2 = RUNX3.13, 3 = RUNX3.53. The coordinates have been obtained using the ‘Blast Like Alignment Tool’ (BLAST).

Information about RUNX3 structure, promoters and amplicons location is available in Figure 1.

**Figure 2:**
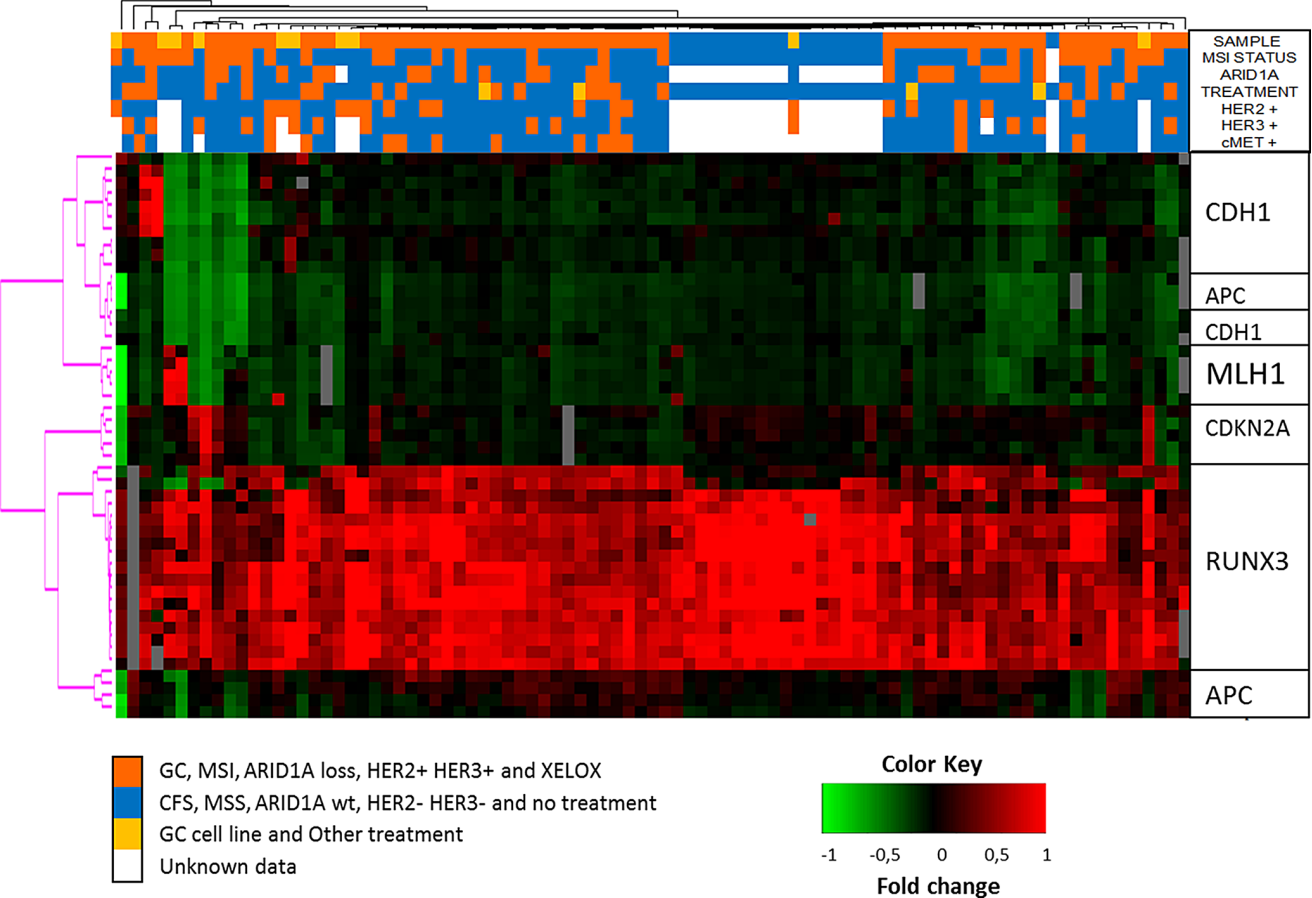
Unsupervised hierarchical clustering of the methylation levels measured in all 47 promoter-CpG islands of 5 GC-related genes See color key in the image.

Unsupervised hierarchical clustering of the methylation levels of all 47 promoter-CpG islands in 5 GC-related genes (Figure 2) revealed no significant methylation-level subgroups between MSI and MSS GC samples or MSI and MSS cell lines. Nevertheless, CFSs clustered together showing higher levels of *RUNX3* methylation compared to GC samples. Additionally, *RUNX3* methylation was also higher than in all the other genes in all of the samples evaluated.

When we compared the average methylation levels between MSI and MSS GC samples, only *MLH1* showed statistically-significant differences associated with MSI status (*p*-value = 0.1 × 10^−6^), although *CDKN2A* and *CDH1* showed a trend towards significance (*p*-values of 0.05 and 0.09, respectively). These results were similar when we compared the MSI and MSS GC cell lines, which confirm the association between hypermethylation of these gene-promoters and MSI status.

**Figure 3:**
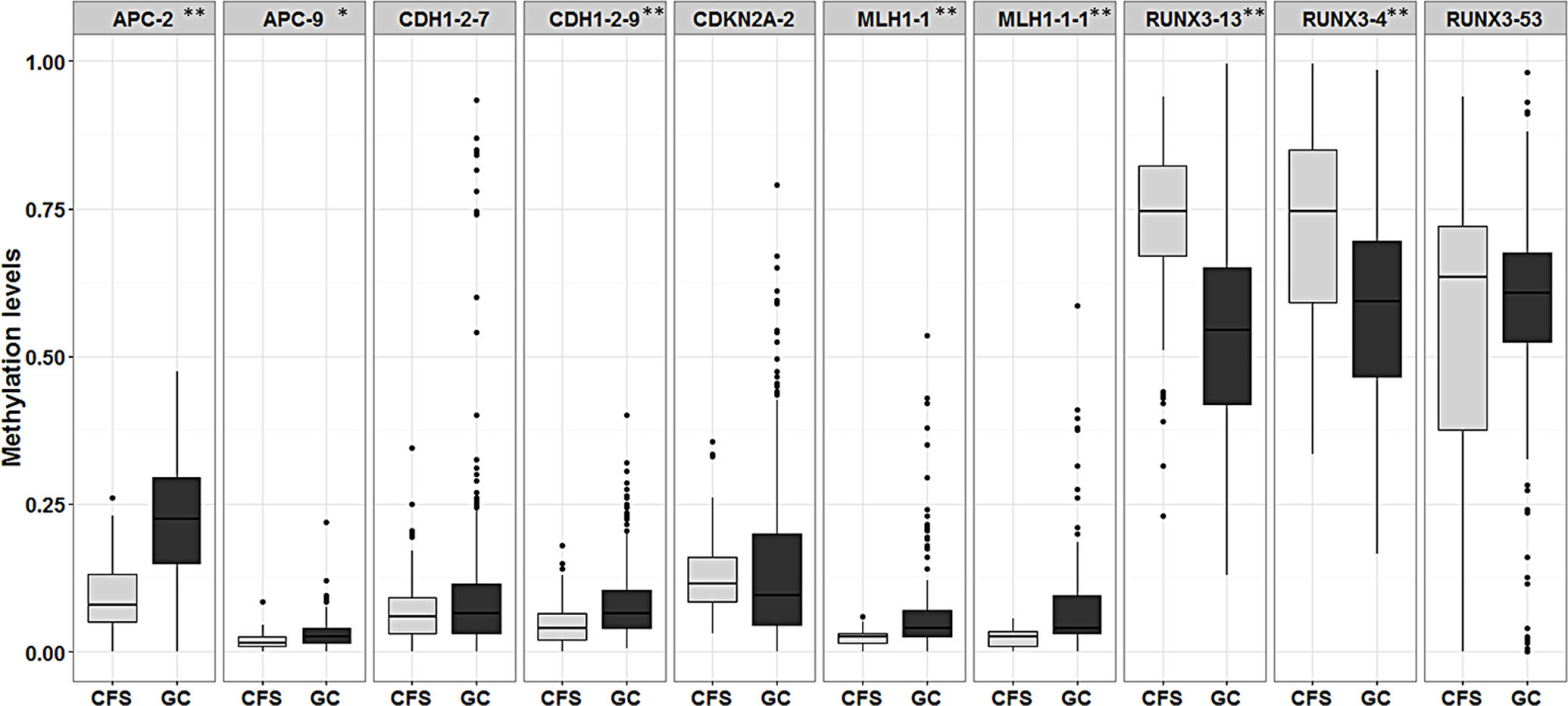
Box plot showing differences in the average methylation of amplicons in gastric cancer (GC) versus cancer-free samples (CFS) * signifies *p*-values < 0.05 while ** signifies *p*-values < 0.01. Average amplicon methylation is represented in light grey for CFSs and in dark grey for GC samples.

Furthermore, comparisons between the average methylation levels in GC samples and CFSs showed statistically significant differences between one or more amplicons in *APC* (APC.2), *CDH1* (CDH1.29), *MLH1* (MLH1.1 and MLH1.11), and *RUNX3* (RUNX3.4 and RUNX3.13), as shown in Figure 3. Surprisingly, the RUNX3.53 amplicon, located proximal to the first exon, showed a trend which was completely opposite to the other *RUNX3* amplicons (4 and 13) located in the P1 sequence, which were both hypermethylated in GC samples compared to CFSs.

**Figure 4:**
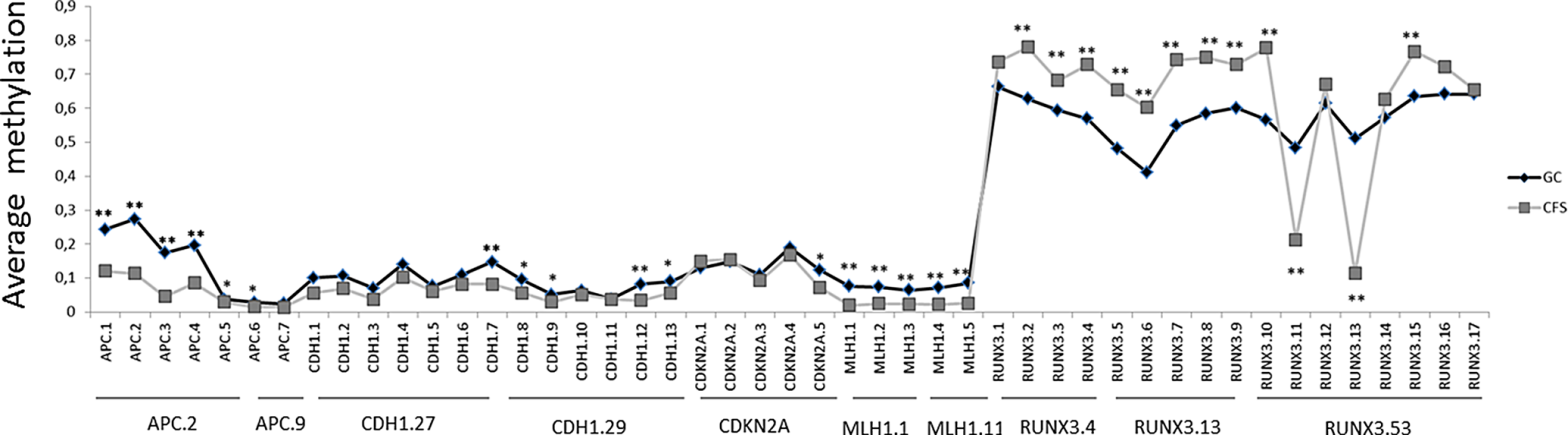
Differences in average amount of methylation in a single CpG island in gastric cancer (GC) versus cancer-free samples (CFS) * signifies *p*-values < 0.05 and ** signifies *p*-values < 0.01. Y axis represents methylation levels. Average CpG methylation is represented in grey for CFSs and in black for GC samples.

Beside these aforementioned results, a total of 29 CpG islands (19 hypermethylated and 10 hypomethylated) spread over 5 genes, showed significant differences in methylation levels (FDR corrected *p*-values) when we compared GC samples with CFSs, as shown in Figure 4. Finally, lower levels of *RUNX3* methylation were correlated with the intestinal GC subtype, according to Lauren classification (*p*-value > 0.001). There were no other associations found and none of the variables studied was correlated with differences in surveillance.

Information about the amplicon sequences and lengths, and their predicted-associated transcription factors (with sequence similarities greater than 0.85) is provided in Supplementary Table 1.

### Immunohistochemical assay

In order to extend our evaluation of *RUNX3* function in GC, we studied RUNX3 protein expression using IHC. We also evaluated ARID1A expression because it seems to play a key role in gastric carcinogenesis and it served as a control reference TSG to compare to *RUNX3,* which has been wrongly categorized as a TSG in the past. Clinicopathological patient features included in the IHC analysis of RUNX3 and ARID1A protein expression are shown in Table 2.

**Table 2:** Characteristics of patients included in the immunohistochemical analysis (*N* = 40)

	*N*	%
**Median age (range)**	75 (49–88)	
**Sex**		
Male	29	72.5
Female	11	27.5
Pathological Stage*		
0–I	20	50.0
IIa	9	22.5
IIb	11	27.5
**Tumor Localization**		
GE junction	1	2.5
Cardias	4	10.0
Fundus	2	5.0
Body	12	30.0
Antrum	20	50.0
Gastrectomy stump	1	2.5
**Histology**		
Intestinal	25	62.5
Diffuse	12	30.0
Both	2	5.0
Unknown	1	2.5
**Grade**		
1	7	17.5
2	15	37.5
3	8	20.0
Unknown	10	25.0
**HER2 Expression**		
Positive = “+++” or FISH positive	9	22.5
Negative = “–“ or FISH negative	30	75.0
Unknown	1	2.5
**HER3 Expression**		
Positive	12	30.0
Negative	27	67.5
Unknown	1	2.5
**Microsatellite Status**		
MSI	11	27.5
MSS	29	72.5

GE, gastroesophageal; MSI, microsatellite instability; MSS, microsatellite stability;

* Pathological staging was based on the TNM classification system for malignant tumors, 7th edition.

### Characterization of the mucosal tissue infiltrate

Analysis of the peritumoral mucosa revealed metaplasia in 50.0%, ulceration in 67.5%, and peritumoral infiltrates in 97.5% of the samples (Table 3). In parallel, for 27.5% of the patients, the cells in the stromal peritumoral infiltrate presented a “predominant lymphocytic phenotype”, while for the remaining 72.5% of the patients, these cells presented a “non-predominant lymphocyte phenotype” (Table 3). Up to 40.0% of the patients showed low or moderate *H. pylori* presence in their mucosal tissue samples, and most of the changes found were located either in the fundus or in the body of the stomach. No statistical associations were found between *H. pylori* infection and *RUNX3* expression. Additional data regarding mucosal changes are shown in Table 3.

**Table 3:** Gastric mucosa changes and *Helicobacter pylori* infection status in patients included in the immunohistochemical analysis

	Frequency (*n* = 40)	%
**Atrophy**		
(−)	12	30.0
(+)	27	67.5
(++)	0	0.0
(+++)	1	2.5
**Dysplasia**		
(−)	15	37.5
(+)	10	25.0
(++)	1	2.5
(+++)	14	35.0
**Hyperplasia**		
(−)	39	97.5
(+)	1	2.5
**Metaplasia**		
(−)	20	50.0
Complete	2	5.0
Incomplete	18	45.0
**Erosion**		
(−)	13	32.5
(+)	21	52.5
(++)	1	2.5
(+++)	5	12.5
**Inflammation**		
(−)	1	2.5
(+)	11	27.5
(++)	6	15.0
(+++)	22	55.0
**Type of cells in peritumoral infiltrate**		
Predominant lymphocyte phenotype	11	27.5
Non-predominant lymphocyte phenotype	29	72.5
***Helicobacter pylori* infection**		
(−)	24	60.0
Low	15	37.5
Moderate	1	2.5

(−) = negative presence of mucosal changes or the absence of *Helicobacter pylori* in the tissue. (+), (++), (+++) = Low, moderate, or high presence of mucosal changes, respectively.

### Analysis of RUNX3 and ARID1A expression

**Figure 5:**
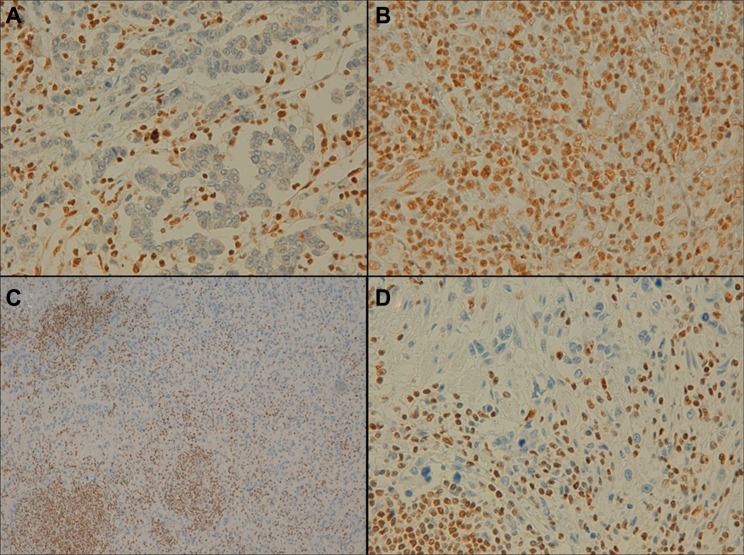
Representative example of ARID1A and RUNX3 expression in gastric epithelial neoplasia and the peritumoral infiltrate (immunohistochemical staining findings) (**A**) Negative expression of ARID1A in the tumor and positive expression in the peritumoral infiltrate (×40 magnification). (**B**) Positive expression in the tumor and in the peritumoral infiltrate (×40 magnification). (**C** and **D**) Negative RUNX3 expression in the tumor and positive expression in the peritumoral infiltrate (×10/×40 magnification, respectively).

Normal gastric mucosa, gastric tumor, and peritumoral tissues were compared for RUNX3 and ARID1A protein expression. Significant differences were found in RUNX3 expression levels between tumor and peritumoral infiltrate (*p*-value < 0.0001) and between the adjacent mucosa and the peritumoral infiltrate (*p*-value < 0.0001) samples. In addition, ARID1A expression was also statistically different between tumor and adjacent mucosa tissues (*p*-value = 0.009) and between tumor and peritumoral infiltrate samples (*p*-value = 0.018). Specifically, ARID1A levels were reduced in 12.5% of the tumor tissues, whereas RUNX3 levels were lower in 87.5% of the tumor tissues analyzed. Adjacent normal gastric mucosa samples presented low protein expression levels for both RUNX3 and ARID1A. Regarding the peritumoral infiltrate, RUNX3 and ARID1A were highly expressed (“+++”) in 90.0% and 92.5% of cases, respectively (Figure 5).

Spearman correlation analysis showed a positive correlation between RUNX3 expression in the peritumoral infiltrate and inflammation (*r* = 0.37), but no other correlations with the infiltrate mucosal tissue remained significant after Spearman correspondence analysis. With regard to microsatellite status, 11 patients (27.5%) presented MSI. Nevertheless, when the mucosal changes, as well as the ARID1A and RUNX3 expression, were compared between the MSI and MSS groups, the differences were not significant. The RUNX3 and ARID1A expression levels are shown in Table 4.

**Table 4:** RUNX3 and ARID1A protein expression (*N* = 40)

Characteristic	*N*	%
IHC RUNX3 expression		
Normal mucosa (−)	38	95
(+)	2	5
Tumor (−)	35	87.5
(+)	4	10
(++)	1	2.5
Peritumoral infiltrate		
(+)	2	5
(++)	2	5
(+++)	36	90
IHC ARID1A expression		
Normal mucosa (−)	0	0
(+)	2	5
(++)	1	2.5
(+++)	37	92.5
Tumor (−)	5	12.5
(+)	5	12.5
(++)	0	0
(+++)	30	75
Peritumoral infiltrate		
(+)	2	5
(++)	1	2.5
(+++)	37	92.5

IHC, immunohistochemistry.

### Clinical outcome

The median overall patient survival was 92 months; and patients with high *ARID1A* expression levels in the adjacent mucosa had significantly better survival times than those with negative, low, or moderate expression levels of this gene (*p*-value = 0.018). There were no other differences in survival between the MSI and MSS groups, or in the outcome when comparing *ARID1A* or *RUNX3* expression in tumoral tissue with that in the peritumoral infiltrate.

### Methylation analysis validation

**Figure 6:**
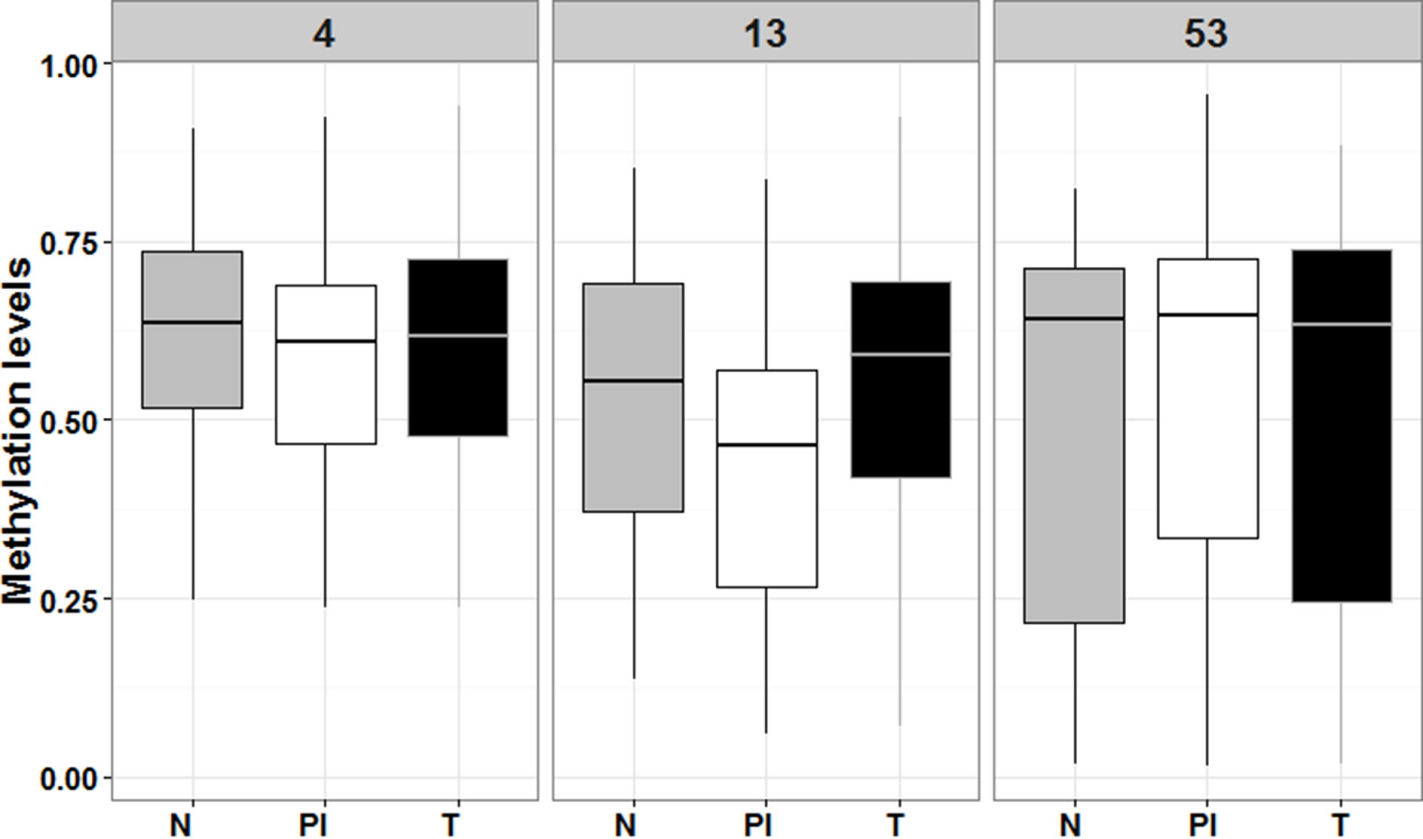
Box plot comparing *RUNX3* levels of methylation among the tree type of microdissected tissues N means normal adjacent mucosa (in grey), PI means peritumoral infiltrate (in white) and T means tumoral tissue (in black). Numbers 4, 13 and 53 refer to RUNX3.4, RUNX3.13 and RUNX3.53 amplicons, respectively.

To validate the relationship found between *RUNX3* hypermethylation in GC and CFS we performed a second methylation analysis, comparing the *RUNX3* methylation status in microdissected tumoral, normal, and peritumoral inflammatory infiltrate tissues. The results showed hypomethylation in peritumoral inflammatory tissues compared to normal and tumoral tissues, although these differences where only statistically significant for the RUNX3.13 amplicon (*p*-value = 0.03) (Figure 6). This finding correlates with our IHC protein analysis results, and seems to corroborate the idea that promoter methylation plays a key role in regulating *RUNX3* expression.

## DISCUSSION

Many methylated genes have been related to gastric carcinogenesis but useful markers that could improve early diagnosis, prognosis, or be used to design better treatments are still not available. The existence of the CIMP, widely described in CRC [17], it is still controversial in GC, and although it has been explored in several studies, a standard panel of methylation markers defining it has not yet been proposed [10, 11]. We searched for changes in the methylation status of five candidate genes in GC samples and showed different promoter methylation levels in the *APC, CDH1, CDKN2A, MLH1,* and *RUNX3* genes. Unlike the others, the latter was hypermethylated in cancer-free samples (CFSs). These findings corroborate the hypothesis that methylation is involved in regulating these genes in GC [7, 8, 11, 16, 18, 28].

Surprisingly, we found no grouping between MSI and MSS samples when we applied unsupervised hierarchical clustering, indicating that the selected genes seem to be not sufficiently good markers (at least not alone) as to represent a CIMP for the MSI group [29, 30]. Thus, further investigation into the potential existence of a CIMP phenotype in GC, as a different subgroup with particular clinical and molecular characteristics is still required to find good markers to discriminate this subgroup of tumors.

*RUNX3* is located in chromosome 1p36, and belongs to the Runt (RUNX) family of related transcription factors also known as alpha-type core-binding factors (CBFαs). *RUNX3* was believed to be a tumor suppressor gene (TSG), although conflicting results have since emerged [21–23]; it has been implicated in GC pathogenesis and also plays a key role in GC immunity. Its promoter area is divided in two regions of interest (P1 and P2): the P1 region is related to disease progression while the P2 region has not previously been correlated with *RUNX3* silencing or GC progression [22]. Furthermore, aberrant P1 hypomethylation signatures have been associated with early inflammatory, pre-neoplastic, and tumor stages [24]. Thus, we examined two amplicons in P1 (RUNX3.4 and RUNX3.13), and another proximal to the first exon (RUNX3.53), and were able to corroborate the principal role of P1 in gene-expression control. Additionally, the inverse results found for amplicon RUNX3.53 compared to the other amplicons located in the P1 sequence raises the question of whether the P1 region is exclusively involved in *RUNX3* regulation, especially given that the other CpG-rich zones are not yet fully understood and have not been clearly related to disease progression.

To further investigate the role of *RUNX3* in GC, we performed IHC analysis which identified marked RUNX3 expression in the peritumoral inflammatory infiltrate, but almost no expression in tumoral or normal adjacent mucosa, in over 90% of the samples analyzed. Methylation analysis of a validation cohort of 14 samples, including immune infiltrate, tumoral, and normal adjacent mucosa tissues revealed decreased methylation levels in the immune infiltrate compared to the other two tissue types. This result supports the idea that *RUNX3* promoter hypermethylation acts as a silencing mechanism in normal and tumoral tissues, and thus the important role of this gene in immunological cells. Similar findings were found by Kurklu et al. in 2014 in a comprehensive study of *RUNX3* methylation which clearly showed it is silenced in tumoral and normal tissue and is overexpressed in every type of immune cell [15].

More than a decade ago *RUNX3* was catalogued as a major TSG in GC and in other cancers. However, new evidence has subsequently emerged showing that *RUNX3* is not expressed in normal gastric and other epithelia which has challenged this *RUNX3*-TSG paradigm [15, 22]. There is still controversy about the role of *RUNX3* in gastric carcinogenesis, but it seems that this gene may have important functions in immunity and inflammation and thereby might indirectly influence epithelial tumor development through aberrant P1 hypomethylation [15]; our data further reinforce this as a silencing mechanism in normal and tumoral tissues.

Most of our patients presented a “non-predominant lymphocyte phenotype”. Gajewski et *al*. described two mechanisms of immune signaling, depending on the predominant cells in the peritumoral infiltrate. In T cell-infiltrated tumors, chemokines support the influx of CD8+ effector T cells, but these subsequently become functionally inhibited by the effects of PD-L1, IDO, regulatory T cells, and anergy. The development of this phenotype appears, in part, to be promoted by type I interferon signaling and CD8a+ dendritic cells. In non-T cell-infiltrated tumors, there is poor chemokine expression and lack of T cell infiltration but there is also a minimal presence of defined immune inhibitory pathways and it has been speculated that these tumors also have denser stroma and alternative myeloid or macrophage populations. Although this distinction has been best characterized in patients with melanoma, similar immune phenotypes may be operational in other subsets of solid tumors, some of which show T cell infiltration. T cell-infiltrated tumors may optimally respond to therapies targeting immune system inhibitory mechanisms, whereas non-T cell-infiltrated tumors may require additional interventions to help promote optimal inflammation and innate immune activation in the tumor microenvironment [31]. One limitation of our study is that we performed morphological characterization of the immune peritumoral infiltrate but did not use specific antibody stains to identify the immune cells present. A standardized procedure to immune tumor-microenvironment profiling would be needed in future studies in order to better understand immune cell signaling in specific context.

In order to elucidate the role of *RUNX3* as a TSG we evaluated the expression of *ARID1A,* a well-known TSG; ARID1A expression was lost in 12.5% of the tumor samples, which agrees with previously published results [32, 33]. Furthermore, survival was significantly longer for patients with high *ARID1A* expression in normal epithelium (*p*-value *= 0.018*) which is also in concordance with published data showing that the maintenance of *ARID1A* expression is associated with a good prognosis, whereas its loss is associated with a poor prognosis and is linked with advanced GC [24, 34–36]. No associations were found between RUNX3 or ARID1A and in our clinicopathological variables or between RUNX3 and *H. pylori* infection. However, this might be explained by the small sample size or because *H. pylori* is only detected well in the early phases of GC, while our samples were mostly GC resections conserved in paraffin.

In conclusion, our results showed aberrant promoter methylation in genes related with GC carcinogenesis, specifically in the *APC, CDH1, CDKN2A, MLH1,* and *RUNX3* genes, as well as the presence of a non-lymphocytic-predominant infiltrate with high *RUNX3* expression. Non-lymphocytic infiltrated tumors may require additional interventions aimed at promoting optimal inflammation and innate immune activation in the tumor microenvironment, perhaps by increasing *RUNX3*-mediated inflammatory signaling.

## MATERIALS AND METHODS

### Patient selection and data collection

Between January 2003 and December 2013, we obtained 220 samples from consecutive, non-related patients diagnosed with sporadic GC and 18 gastric tissue samples from cancer-free patients (CFS) at the Medical Oncology Unit in the INCLIVA Biomedical Research Institute in Valencia, Spain. Samples where formalin-fixed paraffin-embedded (FFPE) conserved and were evaluated for their tumor content; sections containing more than 30% tumor cells were selected by an expert pathologist. Most patients included had not received chemotherapy prior to surgery, although some had been treated with FOLFOX (folinic acid, fluorouracil and oxaliplatin) or XELOX (capecitabine plus oxaliplatin). Clinicopathological and follow-up information was retrieved for all of the participants. All the study subjects gave their written informed consent, and the study protocol was approved by the Ethics Board at the INCLIVA Biomedical Research Institute.

### DNA isolation

Genomic DNA was isolated from FFPE tissues from four unstained 20 μm-sections using a QIAamp DNA FFPE tissue kit (QIAGEN). The DNA concentration was quantified in samples using a NanoDrop spectrophotometer (NanoDrop Technologies, Wilmington, DE, USA) and subsequently stored at −20°C.

### Methylation analysis

The methylation status of 47 promoter-CpG islands in the *APC, CDH1, CDKN2A, MLH1,* and *RUNX3* genes was studied using an EpiTYPER assay for high-throughput analysis of DNA methylation patterns (Sequenom, San Diego, CA, USA), in 35 microsatellite stable (MSS) GC and 26 MSI samples, 18 CFSs, and 6 MSS and 4 MSI cell lines. In the case of *RUNX3*, two amplicons (4 and 13) in the promoter 1 (P1) sequence, and another one in a CpG-dense zone proximal to exon 1 and upstream of the promoter 2 (P2) sequence, have been examined, in order to stablish possible differences in regulation between them.

In brief, in this method bisulfite-converted DNA is amplified by T7-promoter-tagged PCR, followed by generation of a single-stranded RNA molecule and subsequent base-specific cleavage by RNase A. The mixture of cleavage products (differing in length and mass) are analyzed by matrix-assisted laser desorption/ionization time-of-flight mass spectrometry (MALDI-TOF-MS). Differences in the template-DNA methylation profile result in changes in the nucleotide sequence after bisulfite treatment, which in turn yields different fragment masses in the assay. The abundance of each fragment (signal/noise level in the spectrum) is indicative of the amount of DNA methylation in the interrogated sequence [37–39].

For the *RUNX3* methylation validation analysis, micro-dissected and punched paired tumor, normal adjacent mucosa, and peritumoral infiltrate tissues were evaluated in 14 samples, using the same protocol described above.

### Immunohistochemistry assays

Immunohistochemistry (IHC) assays were performed in 40 TNM stage I-II patients; gastric tumor, adjacent non-cancerous mucosa, and peritumoral infiltrate tissues were histologically characterized for all of them. *ARID1A* and *RUNX3* expression was evaluated using an anti-*ARID1A* polyclonal rabbit antibody (HPA005456, dilution 1:500, Sigma-Aldrich) and an anti-*RUNX3* monoclonal mouse antibody (R3-5G4, dilution 1:200, ABCAM), as previously described [32]. Microsatellite status was determined by analyzing MLH1, MSH2, PMS2, and MSH6 using primary antibodies against each protein according to the manufacturer's recommendations (DAKO, Glostrup, Denmark). Tumors were regarded as positive for ARID1A or RUNX3 if the tumor cells showed nuclear immunoreactivity; adjacent mucosa and peritumoral infiltrate tissues were regarded as positive if epithelial and inflammatory cells both showed nuclear immunoreactivity. Mucosal morphological tissue changes (classified as low, moderate, or severe atrophy and dysplasia), as well as the immune-predominant phenotype, were also determined based on hematoxilin and eosin (H&E) staining. For microsatellite status, total lack of immunoreactivity was classified as a loss of protein expression and was considered as evidence of MSI. The apparently normal adjacent tissue was used as an internal control.

HER2, HER3, and cMET expression data were determined via routine hospital protocols and were available for all the patients in our database.

Inflammation was evaluated by morphological criteria without IHC staining. Diffuse infiltration or follicular aggregates of lymphocytes and plasma cells around the tumor infiltration line were analyzed in H&E-stained sections at 10× magnification by an expert pathologist. Inflammation was scored as “+”, “++”, or “+++”, according to the grade of the infiltrate identified. The predominant cell type in the peritumoral infiltrate was also recorded and classified as “Predominant lymphocyte phenotype” or “non-predominant lymphocyte phenotype” according to the presence of 50% or more lymphocytes in the peritumoral infiltrate in sections observed at 10× magnification.

### Histochemical identification of *Helicobacter pylori*

The presence of (*H. pylori*) was monitored using the Warthin-Starry technique [40] with Dako's Artisan Link pro automatic system, as per the manufacturer's recommendations (Dako, Glostrup, Denmark).

### Microsatellite instability determination by PCR

The Type-it Microsatellite PCR kit (Qiagen, Hilden, Germany) was used to co-amplify five markers (NR27, NR21, NR24, BAT25 and BAT26) in a standard multiplex PCR. The PCR conditions were: denaturation at 95°C for 5 minutes, 28 cycles of denaturation at 95°C for 30 seconds, annealing at 60°C for 90 seconds, and extension at 72°C for 30 seconds, followed by a final extension phase at 60°C for 3 minutes. The PCR products were denatured and separated by capillary electrophoresis using an ABI PRISM 310 DNA sequencer and were further analyzed with GeneMapper 3.5 software (Applied Biosystems, Paisley, UK). MSI status was confirmed when two or more markers presented instability and microsatellite stable (MSS) status was confirmed when one or none of the markers presented instability.

### Statistical analysis

Two-way hierarchical clustering analysis, based on the average-linkage clustering algorithm, was performed on the 61 GC samples, 18 CFSs, and 10 GC cell lines, using Gene Cluster and Treeview software (http://www.eisenlab.org/eisen/). Association between clinicopathological and molecular features was analyzed using the Chi-squared test for categorical variables and the Mann-Whitney *U* test for the continuous age variable.

The *t*-test for sample independence was used to study the correlation between the average gene, amplicon, or CpG methylation levels with clinicopathological and molecular features, and to compare differences in methylation levels between MSI or MSS samples and cell lines, and between GC and CFS samples. Survival curves were calculated using the Kaplan-Meier method, and were compared by univariate analysis using the log-rank test. In the case of RUNX3 and ARID1A expression a multivariate COX-regression analysis was performed, in order to test a possible combined effect in survival. The TFBIND online tool (http://tfbind.hgc.jp/) was used to predict possible transcription factor binding sites in the studied regions *in silico*. The *t*-test for sample relatedness was also used to compare categorical RUNX3 and ARID1A protein and gene expression in different tissue types and correlation analyses were performed using Spearman's correlation test; all analyses were performed using SPSS v.19.0 (SPSS, Chicago, IL, USA), POMELO II online tool (http://pomelo2.iib.uam.es/), and R Studio software (http://www.rstudio.com/). A *p*-value of less than 0.05 was considered to be statistically significant in all cases and *p*-values were adjusted for multiple comparisons using the Benjamini & Hochberg false discovery rate (FDR) when we compared methylation levels between single CpGs.

## SUPPLEMENTARY MATERIALS TABLES




