# Supplementary material for: Epigenetic changes in localized gastric cancer: the role of RUNX3 in tumor progression and the immune microenvironment
Source: Oncotarget. 2016 Aug 23;7(39):63424–36. doi: 10.18632/oncotarget.11520 (PMC5325374; doi:10.18632/oncotarget.11520)
Supplement: Supplementary file 1 [file oncotarget-07-63424-s001.pdf]

Epigenetic changes in localized gastric cancer: the role of RUNX3 in tumor progression and the immune microenvironment

Supplementary Materials

Supplementary Table S1: Information about the amplicons used and predicted associated transcription factors (similarity > 0.85). See Supplementary_Table_S1
