# Supplementary material for: Epigenetic changes in localized gastric cancer: the role of RUNX3 in tumor progression and the immune microenvironment
Source: Oncotarget. 2016 Aug 23;7(39):63424–36. doi: 10.18632/oncotarget.11520 (PMC5325374; doi:10.18632/oncotarget.11520)
Supplement: Supplementary file 2 [file oncotarget-07-63424-s002.docx]

| **Supplementary Table 1**. Information about the amplicons used and predicted associated transcription factors (similarity > 0.85) | | | | |
| --- | --- | --- | --- | --- |
| Gene (AMPLICON) | | Amplicon sequence | Length (bp) | Associated transcription factors |
| APC | APC(2) | CTATCTTATGAGCCCTTGTGGGGCTGGGACAGAATTTTATTCATCTTTCTATCATCAGCGTCTAGTACGGGGAGTAGCAAATAGTGAGCACTCGATAGATGTTTGCGGAATAATGGACTAGTGTGTGCAGAAGGATCTATTAACTGGGC | 149 | P300, NKX25  CDPCR1, GATA1, CEBPA, GATA2, CDPCR3HD, CP2  GC, HLF, CDPCR3, GATA3  HNF3B, MZF1, PBX1, HSF1, CAP  HSF2, MYB, SRY, CDXA |
|  | APC(9) | GCAGCAGCCAGGAAAGGTGGAGGACGGCGGGCGTCGCGACCCCCATCCACTGCGTGAGAATGGAGGACCTGCAAAGCCAGGGCCAACTGGAGGCCTGAATGGGGAGTGCGGGGAGGGGAGAGGGTGAGACATGGAGAGAAGAGAGCCAC | 149 | GATA1, SP1, GATA3, MYOD,  CP2, GATA2, CDPCR3HD, P300  AP1, LMO2COM  MZF1, MYB |
| CDH1 | CDH1(27) | GCTGATTGGCTGAGGGTTCACCTGCCGGCCACAGCCAATCAGCAGCGCGGACCCCTCCCCAGGGCGGAGCTGACGGCCCGCCCACCCGGCCTCGCATAGACGCGGTGACCCTCTAGCCTGGAGTTGCTAGGGTC | 134 | RORA1, AP2, LYF1, AP1, GC, SP1, AP4, MZF1,  CP2 |
|  | CDH1(29) | GGGCTGGAGTCTGAACTGACTTCCGCAAGCTCACAGGTGCTTTGCAGTTCCGACGCCACTGAGAGGGGGTGCGTGGCTGCAGCCAGGTGAGCCCCGGAGGCACCGCCCCCCGTACCGCTGATTGGCTGAGGGTTCACCTG | 140 | NFKAPPAB, CETS1P54, AP2, GATA3, USF, NFKAPPAB50, SMZF1, E2, AP4, AML1, SP1, NFY_C, P300, GATA2, MYOD, CAP, GC, DELTAEF1, GATA1, CAAT |
| CDKN2A | | CTAGAAGACCAGGTAGGAAAGGCCCTCGAAAAGTCCGGGGCGCATTCGGCACTTGTTTTGTTTGGTGTGATTTCGTAAACAGATAATTCGTCTCTAGCCCAGGCTAGGAGGAGGAGGAG | 119 | CEBPB, RREB1, AML1, GATA2, GATA1, CEBPA, P53, GATA3, LMO2COM, GC, MZF1, CREBP1, MYOD, HSF1, CAP, LYF1, HSF2, HNF3B, SRY, NKX25, OCT1, PADS, PBX1 |
| MLH1 | MLH1(1) | CTGGCATTCAAGCTGTCCAATCAATAGCTGCCGCTGAAGGGTGGGGCTGGATGGCGTAAGCTACAGCTGAAGGAAGAACGTGAGCACGAGGCACTGAGGTGATTGGCTGAAGGCAC | 116 | CREB, USF, SP1, GC, AP4, CETS1P54, HLF, GATA3, GATA2, ARNT, NMYC, MZF1, MYCMAX, GATA1, CEBPB, CEBPA, DELTAEF1, NFY_C, AML1,  SRY, LMO2COM, CAAT |
|  | MLH1(11) | TGGCCTCCTTCACTCCTGAAGAGAGAGCTGCTCGTGCAGGCCTCGGGCTCTGCCGCCTCTTGGCCTTGAAGGGTCGCGGCTGGGGTGAGGGGGACTTTGTATACCAGTGC | 110 | AP4, LYF1, SP1, ZID, HSF1, SRF_C, AML1, TST1, CDXA, MZF1, CP2 |
| RUNX3 | RUNX3(4) | GAGGTGGCATGGCAGGGAGCTCTCCGGCCGCTGGTGGATCCGGGCTCTGGGCACTCGGTGAGGGGCCCGCGGGGCTCCTAGCCCGCCCAGGCCAATGCTGGCCTTAATTAAGAAGG | 116 | OCT1, HNF3B, CP2, EGR2, AP2, NGFIC, S8, CAAT, CAP, SP1, NKX25 |
|  | RUNX3(13) | TCCAAAAAGAGAGGCAGCCACAAGATCTTCTAAAAGGCCGTGACATCACGGCCCAGGTGACCGCGGCCCAGCCAATGAGCCAAGGCCGCGAGCAGGCTTCTCGCATCCTGTGAGCTGAGGTTGGGT | 126 | USF, AP2, AP4, NF1, GATA2, SP1, CREB, P53, GATA1, DELTAEF1, CAP, CETS1P54, AML1, ELK1, PADS, NFY, CAAT |
|  | RUNX3(53) | CCCAGGGCCCTGGGCTATTGTTACTCACCGCGGATGAAGGTCGGCGAGTAGGTCGGGAAGGAGTCGAAGATGCTGTTCGATGCCATGCCCCGCTCTGAAGAAGGCGAGAATTTTCAGCCCTTCAGGGGGTT | 131 | MZF1, GC, USF, LYF1, HSF1, CHOP, AP2, HSF2, CDPCR3HD, P53, IK2, GATA1, GATA2, SP1, CAP |
|  | bp, base pairs. The RUNX3.4 and RUNX3.13 amplicons are located in the RUNX3 promoter 1 sequence. | | | |
